# In silico screening of non-synonymous SNPs in human TUFT1 gene

**DOI:** 10.1186/s43141-023-00551-4

**Published:** 2023-10-06

**Authors:** Athira Ajith, Usha Subbiah

**Affiliations:** Human Genetics Research Centre, Sree Balaji Dental College and Hospital, Bharath Institute of Higher Education and Research, Chennai, 600 100 Tamil Nadu India

**Keywords:** SNPs, TUFT1 gene, In silico analysis

## Abstract

**Background:**

Tuftelin 1 (*TUFT1*) gene is important in the development and mineralization of dental enamel. The study aimed to identify potential functionally deleterious non-synonymous SNPs (nsSNPs) in the *TUFT1* gene by using different in silico tools. The deleterious missense SNPs were identified from SIFT, PolyPhen-2, PROVEAN, SNPs & GO, PANTHER, and SNAP2. The stabilization, conservation, and three-dimensional modeling of mutant proteins were analyzed by I-Mutant 3.0, Consurf, and Project HOPE, respectively. The protein–protein interaction using STRING, GeneMANIA for gene–gene interaction, and DynaMut for evaluating the impact of the mutation on protein stability, conformation, and flexibility.

**Results:**

Eight deleterious nsSNPs (E242A, R303W, K182N, K123N, R117W, H289Q, R203W, and Q107R) out of 304 were found to have high-risk damaging effects using six in silico tools*.* Among them, K182N and K123N alone had increased stability, whereas E242A, R303W, R117W, H289Q, Q107R, and R203W exhibited a decrease in protein stability, based on DDG values. Meanwhile, all the eight deleterious nsSNPs altered the size, charge, hydrophobicity, and spatial organization of the amino acids and predominantly had alpha helix domains. These deleterious variants were located in highly conserved regions except R203W. Protein–protein interaction predicted that TUFT1 interacted with ten proteins that are involved in enamel mineralization and odontogenesis. Gene–gene interaction network showed that *TUFT1* is involved in physical interactions, gene co-localization, and pathway interactions. DynaMut ΔΔG values predicted that five nsSNPs were destabilizing the protein, ΔΔG ENCoM values showed a destabilizing effect for all mutants, and seven nsSNPs increased the molecular flexibility of TUFT1.

**Conclusion:**

Our study predicted eight functional SNPs that had detrimental effects on the structure and function of the *TUFT1* gene. This will aid in the development of candidate deleterious markers as a potential target for disease diagnosis and therapeutic interventions.

## Background

The genotypic and phenotypic variation between individuals arises through genetic mutation. The genetic variation provides the diversity within and across populations. The source variance in a genome known as single-nucleotide polymorphism (SNP) is the most abundant genetic variation in the human genome [1]. They can modify protein function and serve as important markers for understanding diseases [2]. Among these SNPs, non-synonymous SNPs (nsSNPs) occurring in the functional exonic regions result to changes in amino acid composition. These mutations have detrimental effects such as reducing protein solubility or destabilizing protein structure which affects the protein function. They can influence gene regulation by affecting transcription and translation processes [3].

TUFT1, an acidic protein highly conserved and located on chromosome 1q21-31 with 13 exons and a phosphorylated glycoprotein of 390 amino acids, was initially discovered and sequenced from a complementary DNA library enriched in bovine ameloblasts. They are involved in the development and maturation of extracellular enamel which leads to the mineralization of the epithelial tissue of the vertebrate teeth [4]. It is associated with diseases like amelogenesis imperfecta (AI) and dental caries. AI is the most common hereditary defect in enamel formation. The main structural proteins involved in enamel formation are amelogenin, tuftelin, enamelin, and ameloblastin. The mutation in the genes coding for these structural proteins is known to be associated with different types of AI [5]. They play a vital role in dental enamel mineralization and are implicated in caries susceptibility. Studies showed a positive association between genetic variation in the enamel proteins and higher caries experience [6]. TUFT1 is also involved with adaptation to hypoxia, mesenchymal stem cell function, and neuronal differentiation associated with neurotrophin nerve growth factor. The structural constituent of the tooth enamel includes tuftelin. They are secreted at the early stage of enamel formation and present in extracellular enamel associated with the crystal component. *TUFT1* is expressed in the morula, embryonic stem cells, and soft tissues, such as brain neurons, testis, suprarenal gland, liver, kidneys, and tumor cells [7, 8]. It is found that *TUFT1* expression induced by human HepG2 and MCF-7 cell lines when treated with 1% O_2_ in the hypoxic environment causes tumorigenesis [9].

A study reported one nonsynonymous mutation in exon 1 of *TUFT1* by mutation analysis associated with high caries experience in Turkish samples [6]. Previous epidemiological studies have shown that the association between caries susceptibility and genetic variations at *TUFT1* is involved in the enamel [10]. The TUFT protein in the developing enamel is a candidate gene involved in inherited enamel defects. Considering the above facts, the presence of SNPs in *TUFT1* can be able to influence its expression and functions. This study aims towards examining the potential effect of nsSNPS in TUFT1 protein using a computational approach and screening deleterious nsSNPs by in silico method for further analysis.

## Material and methods

### Retrieving nsSNPs

nsSNPs of the *TUFT1* gene were obtained from the National Center for Biotechnology Information (NCBI) dbSNP database (http://www.ncbi.nlm.nih.gov/snp/).the). SNPs of *TUFT1* were also retrieved from the ENSEMBL database. The TUFT1 protein primary sequence (UniProt accession number: Q9NNX1) was retrieved from the UniProt database.

### Prediction of deleterious nsSNPs by different bioinformatics tools

The effects of nsSNPs on the *TUFT1* gene were analyzed using the following bioinformatics tools: SIFT and PolyPhen-2 were used to predict the deleterious nsSNPs. To increase the accuracy of the in silico approaches and for prioritizing deleterious nsSNPs, nsSNPs that were found to be deleterious by SIFT and PolyPhen-2 were further analyzed by PROVEAN, SNPs & GO, PANTHER, and SNAP2 tools.

#### SIFT (Sorting Intolerant from Tolerant)

SIFT (https://sift.bii.a-star.edu.sg/) [11] is a power tool used to determine whether a change in amino acid substitution alters the protein function based on sequence homology and the physical characteristics of amino acids. The rsIDs of nsSNPs from NCBI’s dbSNP database were submitted as query sequences to SIFT and multiple alignment information was used to analyze tolerated and deleterious substitutions in every position of the query sequence. The result provides nsSNPs as deleterious or tolerated with a SIFT score. A score ≤ 0.05 indicates deleterious and those > 0.05 indicates tolerated. The deleterious nsSNPs were further analyzed to identify the damaging ones.

#### Polyphen-2 server (polymorphism phenotyping v2.0)

PolyPhen-2 (http:// genetics.bwh.harvard.edu/pph2/) [12] is an online tool that predicts the effects of amino acid substitutions on the structure and function of the protein using structural information and multiple sequence alignment. The results are shown as “PROBABLY DAMAGING” with a score of 0.9—1, “POSSIBLY DAMAGING” with a score of 0.4–0.8, or “Benign.”

#### Provean server (Protein Variation Effect Analyzer)

The biological impact of an amino acid substitution on a protein was predicted using the PROVEAN software tool (http://provean.jcvi.org/index.php) [13]. It predicts the damaging effect of protein variation in in-frame insertions, deletions, and multiple amino acid substitutions other than single amino acid substitutions. The default threshold in the results provided by the software is − 2.5, that is variants with a score ≤  − 2.5 are considered “deleterious” while scores >  − 2.5 are considered “neutral.”

#### SNPs&Go server

The disease relationship with the studied SNPs was analyzed using this online web server (http://snps.biofold.org/snps-and-go/snps-and-go.html) [14]. The result is based on the combination of Panther result, PHD-SNP result, and SNPs&GO result. It predicts whether the mutation is disease-related or neutral, the reliability index (RI), and disease probability.

#### PANTHER (Protein Analysis Through Evolutionary Relationship)

PANTHER (https://www.pantherdb.org/tools) [15] was used to evaluate the nsSNP’s functional impact on the protein based on their position-specific evolutionary relationship. FASTA sequence and amino acid changes were included in the input query.

#### SNAP2 (screening for non-acceptable polymorphisms)

SNAP2 (https://rostlab.org/services/snap2web) [16] predicts the functional effects of nsSNPs based on a machine learning tool called a neural network that incorporates evolutionary data, expected secondary structure, and solvent accessibility. The FASTA sequence of *TUFT1* was provided as the input query.

### Analysing the effect on protein stability

I-Mutant server (http://gpcr2.biocomp.unibo.it/cgi/predictors/I-Mutant3.0/I-Mutant3.0.cgi) [17] calculates the protein stability between the wild type and mutant proteins by computing the changes in the Gibbs free energy which can be due to the single amino acid change. This support vector machine utilizes an SVM prediction algorithm to predict protein stability. The energy difference was calculated based on the predicted DDG value. To predict the impact of a mutation on protein stability, the FASTA sequence, the mutation position, and the amino acid change were given as input.

### Evolutionary conservation analysis of nsSNPs

The evolutionary conservation of amino acid positions in a protein molecule was predicted by the Consurf server (https://consurf.tau.ac.il) [18] based on the phylogenetic relationships between homologous sequences. By using an empirical Bayesian method, the predicted evolutionary conservation scores have a confidence interval and are classified as variable (1–4 scores), intermediate (5–6 scores), and conserved (7–9 scores). The FASTA sequence of *TUFT1* was given as the input for identifying the evolutionary conservation of the predicted deleterious nsSNPs.

### Protein secondary structure prediction

The PSIPRED workbench (http://bioinf.cs.ucl.ac.uk/psipred/) [19] makes available several protein annotation tools. The protein structure prediction server PSIPRED was used for secondary structure prediction. The FASTA sequence of the TUFT1 protein was the input format. The server employs an artificial neural network and PSI-BLAST alignment results for protein secondary structure prediction. The MEMSAT-SVM transmembrane topology predictor uses a support vector machine and identifies the transmembrane proteins from the protein sequence as an input and predicts the involvement of the transmembrane helix in pore formation. By using Dompred, PSI-BLAST sequence alignment domain prediction using an *E* value cutoff of 0.01 gives sensitivity and selectivity of domain boundary prediction.

### Prediction of structural effect of nsSNPs

Project HOPE web server (http://www.cmbi.ru.nl/hope/home) [20] was used to predict the structural impact of the nsSNPs of TUFT1. Project HOPE identifies the structural characteristics of the point mutations of the native protein by utilizing the tertiary structure available in the UniProt database and Distributed Annotation System (DAS) servers. We used the protein sequence of TUFT1 as the input.

### Prediction of protein–protein interactions

A pre-computed database STRING (https://string-db.org/) [21] was used to determine protein–protein interactions of TUFT1 to understand the function, structure, molecular action, and regulation of the protein. The protein sequence was used as an input query.

### Prediction of gene–gene interaction

GeneMANIA (https://genemania.org/) [22] is a web interface that utilizes a large collection of functional association data to quickly and accurately detect gene-gene interactions connected to the input gene. Association data consist of protein and genetic interactions, co-localization, co-expression, pathways, and protein domain similarity. GeneMANIA predicted the gene-gene interaction network of the *TUFT1* gene.

### 3D Structure prediction

The 3D structure was predicted using an artificial intelligence system, AlphaFold (https://alphafold.ebi.ac.uk/) [23, 24] which can predict protein structures computationally with accuracy and speed. The UniProt ID of the TUFT1 protein was used as an input to get the alphaFold model.

### Determining the protein stability, flexibility, and interatomic interactions

The structure-based tool DynaMut (http://biosig.unimelb.edu.au/dynamut/) [25] was used to estimate the effect of point mutation on the stability and flexibility of proteins based on interatomic interactions. A mutation list and the wild-type structure in PDB format were given as input. To determine the difference in free energy change (ΔΔG) between the wild-type (WT) and mutant-type (MT) structures, DynaMut uses normal mode analysis (NMA). In addition to its prediction, DynaMut also provides structure-based predictions for mCSM [26], SDM [27], and DUET [28] as well as the ΔΔG prediction of an elastic network contact model (ENCoM) based on NMA. Additionally, DynaMut predicts the mutation as more or less flexible using ENCoM-based difference in vibrational entropy (ΔΔS_Vib_).

## Result

**Fig. 1:**
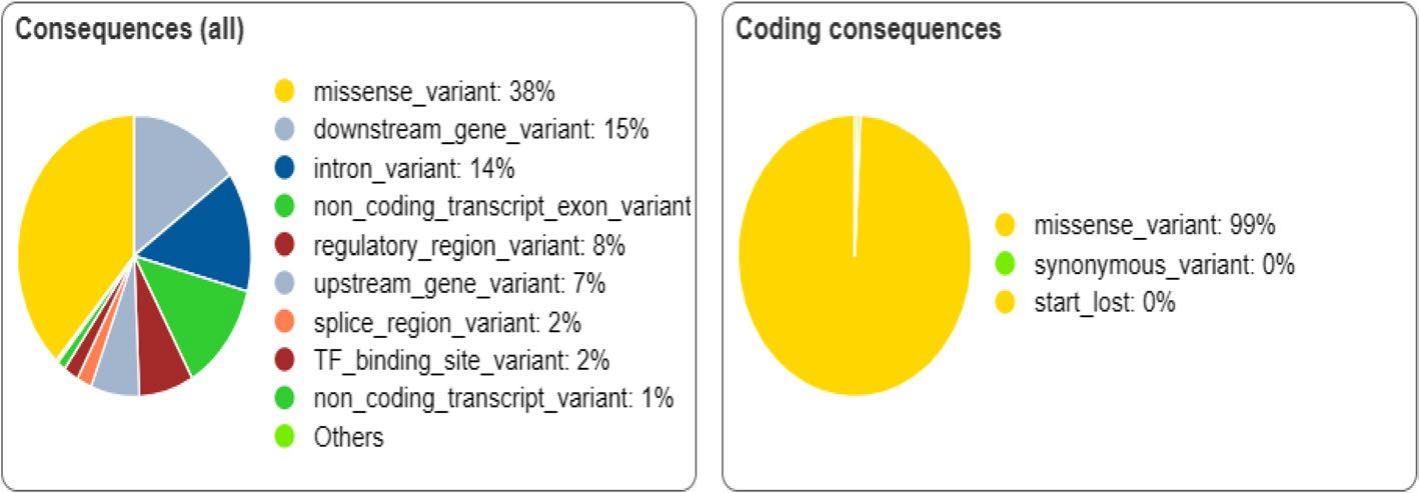
Prediction of *TUFT1* gene by the variant effector predictor of ENSEMBEL

The SNP database in NCBI contains both synonymous and non-synonymous polymorphisms. *TUFT1* gene has a total of 10,860 SNPs, out of which we selected 304 missense nsSNPs for our investigation. Using various in silico prediction tools, we analyzed the deleterious nsSNPs and compared their scores with each tool. Various SNPs of *TUFT1* were predicted using the variant effector predictor of ENSEMBLE as shown in Fig. 1.

### Prediction of deleterious nsSNPs by SIFT program

A total of 304 nsSNPs were selected for SIFT analysis. According to SIFT, the considered deleterious nsSNPs score is 0.05 or below. Among the 304 nsSNPs, 95 nsSNPs were predicted as damaging by SIFT tool whereas the remaining nsSNPs were predicted as “tolerated.”

### Prediction of functional effects of nsSNPs by Polyphen2

The deleterious nsSNPs filtered through the SIFT server were then subjected to the Polyphen server. Out of the 95 nsSNPs, 15 were considered to be “PROBABLY DAMAGING” with a score of 0.9–1, and 24 were observed as “POSSIBLY DAMAGING” with a score of 0.4–0.8. To increase the accuracy of the prediction, the results of both SIFT and Polyphen were combined and these deleterious SNPs of *TUFT1* were considered for further analysis with other in silico tools.

### Prediction of nsSNPs by PROVEAN, SNPs & GO, PANTHER, and SNAP2

The 15 nsSNPs determined by SIFT and Polyphen were subjected to PROVEAN, SNPs&GO, and PANTHER software tools, respectively. Using PROVEAN prediction, 9 nsSNPS were found to be deleterious based on a default threshold score. According to SNPs&GO, 8 nsSNPs were associated with diseases. Moreover, via the PANTHER software tool, 2 nsSNPs were predicted as probably damaging and 13 were probably benign. The SNAP2 tool predicted 4 neutral nsSNPs and 11 nsSNPs were diseases associated. Deleterious and disease-related nsSNPs were investigated further by at least five in silico software. Finally, eight nsSNPs (rs4994616, rs148582735, rs149655288, rs149655288, rs150612239, rs369673392, rs370920800, rs374164451) were identified as the most deleterious and are shown in Table 1.

**Table 1:** Deleterious nsSNP prediction for *TUFT1* by in silico prediction tools

S. no	rs ID	Amino acid position	SIFT (score)	Polyphen 2 (score)	Provean (score)	SNPs & Go RI)	Panther (Pdel)	SNAP2 (score)
1	rs4994616	E242A	Deleterious 0.007	Probably damaging (0.990)	Deleterious (− 4.943)	Disease 2	Probably benign 0.19	Effect 9
2	rs41310883	T175M	Deleterious 0.01	Possibly damaging (0.771)	Deleterious (− 2.692)	Neutral 4	probably benign 0.19	Neutral -12
3	rs140180310	S122N	Deleterious 0.038	Probably damaging (1)	Neutral (− 2.115)	Neutral 3	probably benign 0.27	Neutral -29
4	rs140412170	P376L	Deleterious 0.01	Probably damaging (0.999)	Neutral (− 2.094)	Disease 4	probably benign 0.19	Effect 41
5	rs148582735	R303W	Deleterious 0.001	Probably damaging (1)	Deleterious (− 6.418)	Disease 2	probably benign 0.19	Effect 28
6	rs149655288	K182N	Deleterious 0.023	Possibly damaging (0.954)	Deleterious (− 2.728)	Neutral 5	Probably damaging (0.57)	Effect 32
7	rs149655288	K123N	Deleterious 0.042	Probably damaging (1)	Deleterious (− 2.665)	Neutral 7	Probably damaging (0.74)	Effect 18
8	rs150612239	R117W	Deleterious 0.005	Probably damaging (1)	Deleterious (− 5.596)	Disease 5	probably benign 0.19	Effect 53
9	rs189101009	E93K	Deleterious 0.019	Probably damaging (0.999)	Neutral (-2.276)	Disease 3	probably benign 0.19	Effect 30
10	rs368431369	R386Q	Deleterious 0.021	Probably damaging (1)	Neutral (− 1.549)	Neutral 4	probably benign 0.19	Effect 31
11	rs369673392	H308Q	Deleterious 0.022	Probably damaging (1)	-	-	-	-
12	rs369673392	H289Q	Deleterious 0.025	Probably damaging (0.999)	Deleterious (− 3.341)	Disease 3	probably benign 0.19	Effect 1
13	rs370920800	R203W	Deleterious 0.005	Probably damaging (0.992)	Deleterious (− 4.163)	Disease 2	probably benign 0.19	Effect 21
14	rs373535548	R206Q	Deleterious 0.027	Possibly damaging (0.508)	Neutral (− 1.214)	Neutral 4	probably benign 0.19	Neutral − 24
15	rs374164451	Q107R	Deleterious 0.039	Probably damaging (0.997)	Deleterious (− 2.776)	Disease 0	probably benign 0.19	Effect 14

### Protein stability prediction by I-Mutant 3.0

I-Mutant 3.0 analysis of the nsSNPs revealed that six of the eight deleterious nsSNPs decreased the stability of the TUFT1 protein as shown by its score, which was < 0 for for every mutation. Table 2 displays the free energy change (ΔΔG) values, along with predictions and relative indexes.

**Table 2:** I-Mutant prediction based on DDG value and binary classification

rsID	Aminoacid change	DDG value (Kcal/mol)	Prediction	Relative index (RI)
rs4994616	E242A	− 0.17	Decrease	3
rs148582735	R303W	− 0.06	Decrease	1
rs149655288	K182N	0.29	Increase	7
rs149655288	K123N	0.48	Increase	0
rs150612239	R117W	− 0.42	Decrease	5
rs369673392	H289Q	− 0.19	Decrease	4
rs370920800	R203W	− 0.32	Decrease	4
rs374164451	Q107R	− 0.04	Decrease	4

### Conservation profile of deleterious nsSNPs by ConSurf

**Fig. 2:**
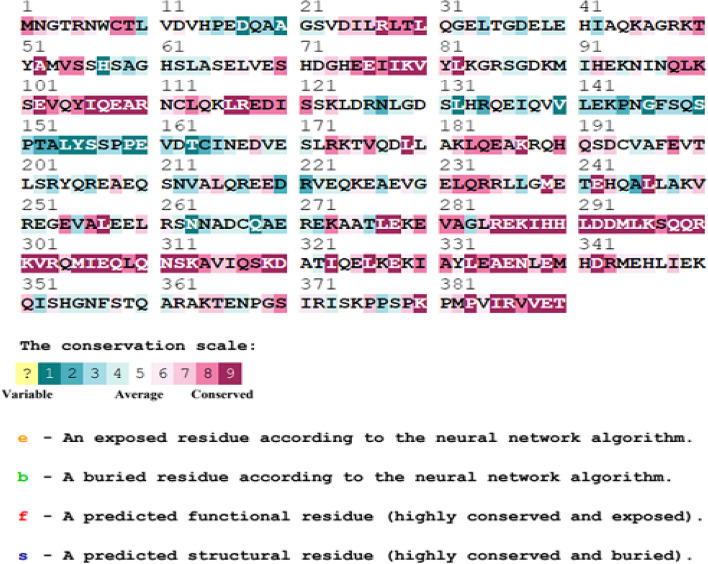
Evolutionary conservation analysis of amino acid residues of TUFT1 by ConSurf. The color-coding bar represents the conservation scheme

The functional, structural, and evolutionary conservancy of amino acid residues of the *TUFT1* were recognized by the ConSurf server (Fig. 2). We found that E242A, R303W, R117W, H289Q, and Q107R are functional residues and highly conserved and exposed. K182N and K123N are conserved and exposed residues but R203W is variable and exposed residue.

### Prediction of secondary structures by PSIPRED server

**Fig. 3:**
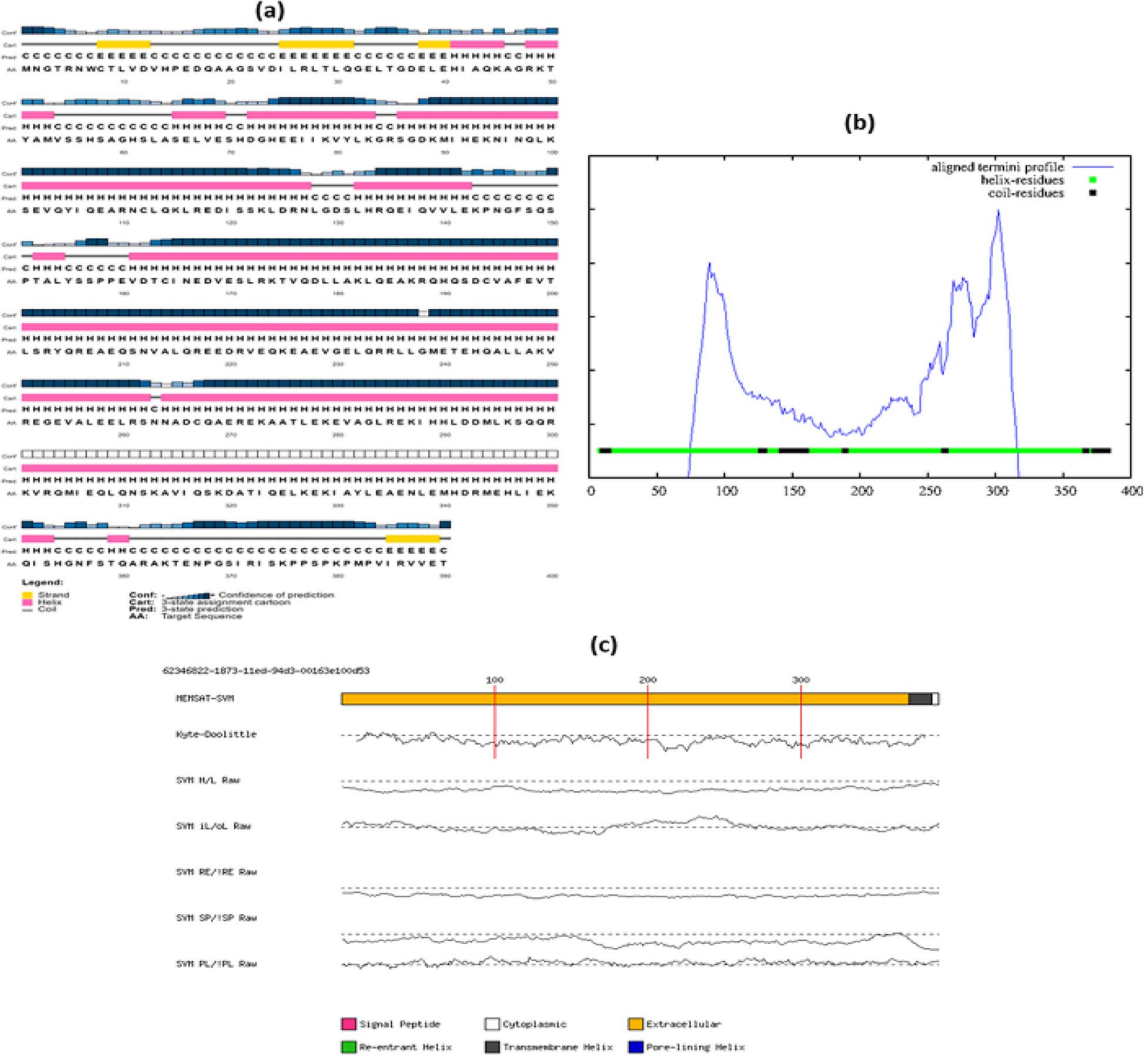
TUFT1 Secondary Structure Prediction using PSIPRED server. **a** Secondary structure showing a helix, coil, and strand. **b** Domain prediction using DomPred. **c** Schematic diagram of the MEMSAT3

The distribution of the alpha helix, beta sheet, and coils in the TUFT1 secondary structure was predicted by PSIPRED. The results revealed a mixed distribution of coil, strand, and alpha helix. As generated by PSIPRED, the helix was shown to be the main secondary structural motif, followed by coil and strand as shown in Fig. 3a. The PSIPRED prediction along with the transmembrane topology and aatypes of the eight deleterious nsSNPs were given in Table 3. The DOMPRED output gives a graph that utilizes the PSI-BLAST aligned termini algorithm. The graph shows secondary structure regions, and peaks in the aligned termini profile represent regions that form a structural domain boundary. The highest peaks in the graph correspond to the putative domain boundaries (Fig. 3b). MEMSATSVM predictions include a prediction of pore-lining helices, and the output was the membrane topology annotated with the predicted helix coordinates (Fig. 3c). All the damaging substitutions are alpha helix, and their transmembrane topology was extracellular and also they are polar.

**Table 3:** Secondary structure prediction of TUFT1 by PSIPRED server

Amino acid change	PSIPRED	MEMSAT3 (transmembrane topology and helix prediction)	aatypes
E242A	Helix	Extracellular	Polar
R303W	Helix	Extracellular	Polar
K182N	Helix	Extracellular	Polar
K123N	Helix	Extracellular	Polar
R117W	Helix	Extracellular	Polar
H289Q	Helix	Extracellular	Polar
R203W	Helix	Extracellular	Polar
Q107R	Helix	Extracellular	Polar

### Structural impact of nsSNPs by Project HOPE

Project HOPE revealed the wild-type and mutant amino acid differences in terms of physicochemical properties such as specific size, charge, hydrophobicity value, location of the conservation, and the impact of variant amino acid residues on the domain. The results are listed in Table 4.

**Table 4:** Physicochemical properties of wild-type and mutant amino acid residues from Project HOPE

Amino acid change	Amino acid Properties	Schematic structure (original (left) and the mutant (right))	Effect
E242A	Size: mutant residue is smaller than the wild-type residue Charge: wild-type residue charge was Negative; the mutant residue charge is Neutral Mutant residue is more hydrophobic than the wild-type residue		The location of the mutation residue is in a domain binding to other molecules and the variant might disturb the function
R303W	Size: mutant residue is bigger than the wild-type residue Charge: wild-type residue charge was Positive; the mutant residue charge is Neutral Mutant residue is more hydrophobic than the wild-type residue		Variant and the wild type are not similar and this lead to the probably damaging the protein. The mutant is located on the domain that binds to other molecules and the residue might disturb this function
K182N	Size: mutant residue is smaller than the wild-type residue Charge: wild-type residue charge was Positive; the mutant residue charge is Neutral		The variant is located near a highly conserved position The mutant is located on the domain that binds to other molecules and the residue might disturb this function
K123N	Size: mutant residue is smaller than the wild-type residue Charge: wild-type residue charge was Positive; the mutant residue charge is Neutral		The variant and the wild type are not similar and based on conservation scores this mutation is probably damaging to the protein
R117W	Size: mutant residue is bigger than the wild-type residue Charge: wild-type residue charge was Positive, the mutant residue charge is Neutral Mutant residue is more hydrophobic than the wild-type residue		The variant is located near a highly conserved position The mutated residue is located in a domain that is important for binding of other molecules. Mutation of the residue might disturb this function
H289Q	Size: mutant residue is smaller than the wild-type residue		The variant is located near a highly conserved position The mutant is located on the domain that binds to other molecules and the residue might disturb this function
R203W	Size: mutant residue is bigger than the wild-type residue Charge: wild-type residue charge was Positive; the mutant residue charge is Neutral Mutant residue is more hydrophobic than the wild-type residue		Your mutant residue is located near a highly conserved position The mutant is located on the domain that binds to other molecules and the residue might disturb this function
Q107R	Size: mutant residue is bigger than the wild-type residue Charge: wild-type residue charge was Neutral; the mutant residue charge is Positive		Your mutant residue is located near a highly conserved position The mutant is located on the domain that binds to other molecules and the residue might disturb this function

### Analysis of protein–protein interaction

**Fig. 4:**
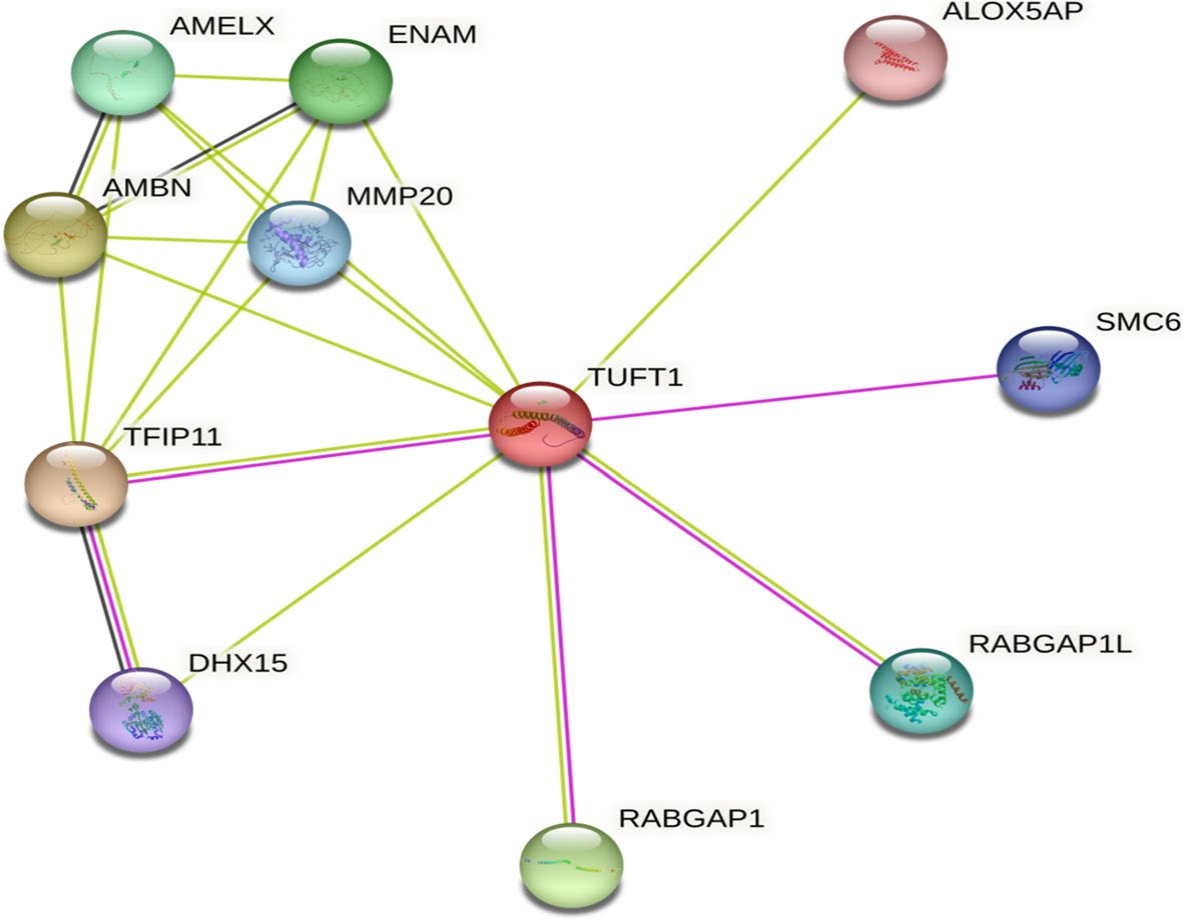
Protein–protein interaction network of TUFT1 using STRING

The STRING network revealed that TUFT1 interacts with 10 proteins which include TFIP11(Tuftelin-interacting protein 11) AMBN (Ameloblastin), RABGAP1 (RabGTPase-activating protein 1), ENAM (Enamelin), AMELX (Amelogenin), RABGAP1L (RabGTPase-activating protein 1-like), MMP20 (Matrix metalloproteinase-20), SMC6 (Structural maintenance of chromosomes protein 6), DHX15 (Pre-mRNA-splicing factor ATP-dependent RNA helicase), ALOX5AP (Arachidonate 5-lipoxygenase-activating protein) (Fig. 4). Except for RABGAP1L and SMC6, the other 8 proteins showed higher interaction based on the confidence score generated by experimental validation and text mining. Due to the nsSNP variants in TUFT1, amino acid alterations may also have an impact on the function of the interacting molecules.

### Analysis of gene–gene interaction

**Fig. 5:**
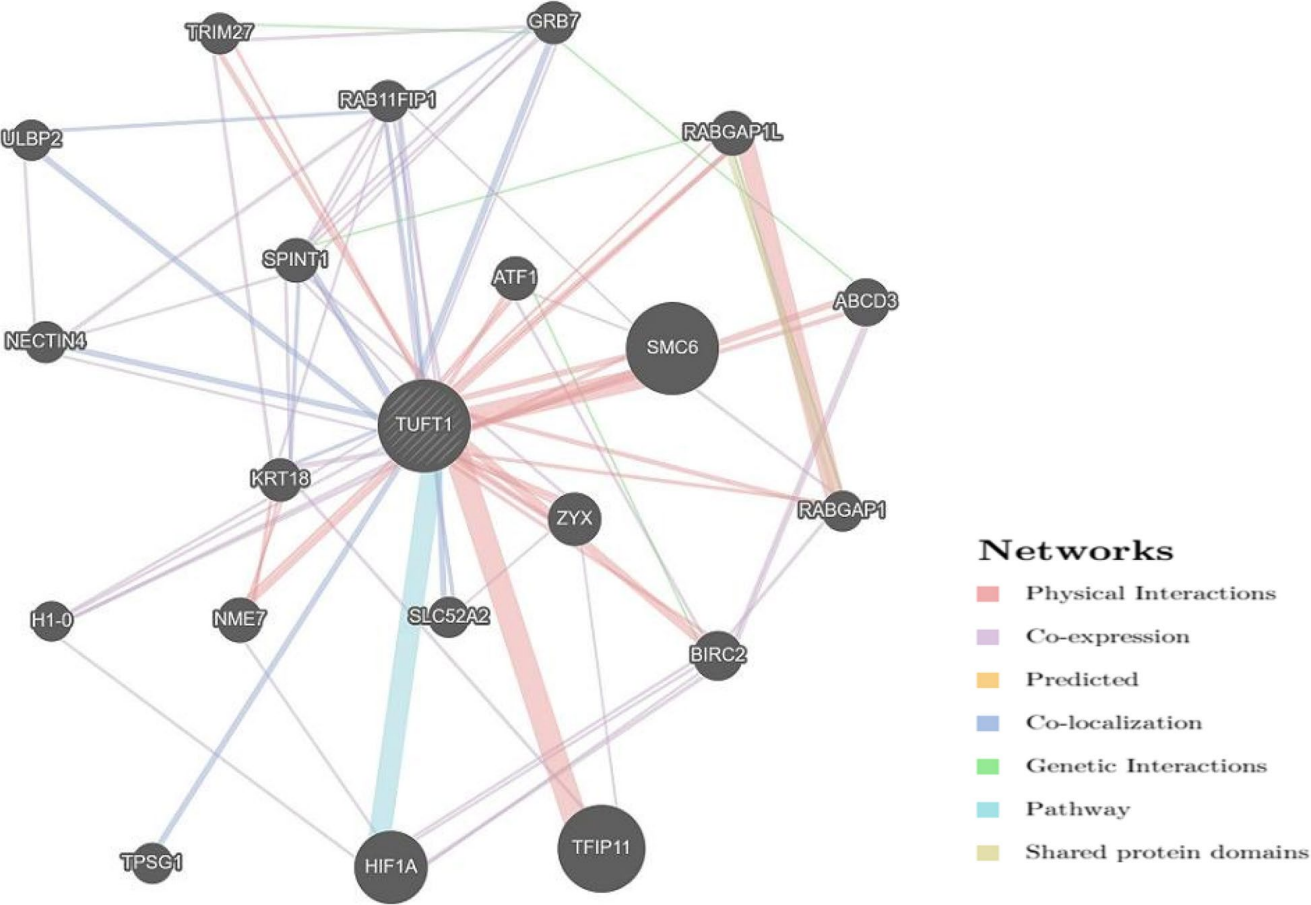
The functional gene–gene interaction network of *TUFT1*

Figure 5 depicts the gene–gene interaction network of the *TUFT1* gene. GeneMANIA revealed that 11 genes had physical interactions, 8 genes co-localize, 1 in pathway interactions, and 2 genes shared a protein domain with *TUFT1.*

### 3D Structure prediction by AlphaFold

**Fig. 6:**
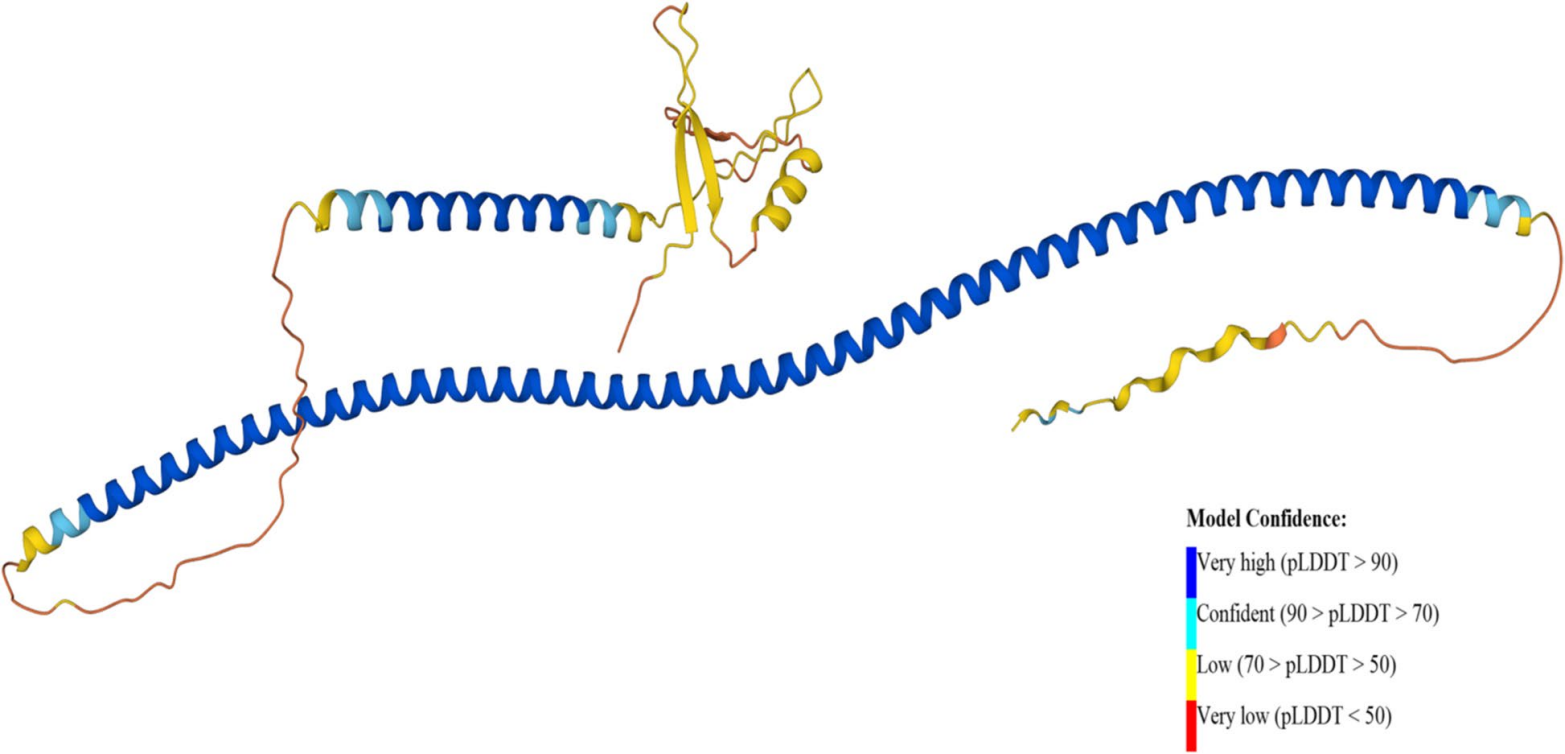
AlphaFold structure of TUFT1 (Uniprot accession number: Q9NNX1)

An individual residue confidence score (pLDDT) between 0 and 100 is generated by the AlphaFold algorithm. The majority of the 3D structural region corresponds to α-helical domains and has extremely high confidence (pLDDT > 90). The remaining components of the model are depicted as unresolved loops with low (70 > pLDDT > 50) and extremely low (pLDDT 50) scores (Fig. 6).

### Predicting the impact of TUFT1 nsSNpson protein conformation, flexibility, and stability by DynaMut

**Fig. 7:**
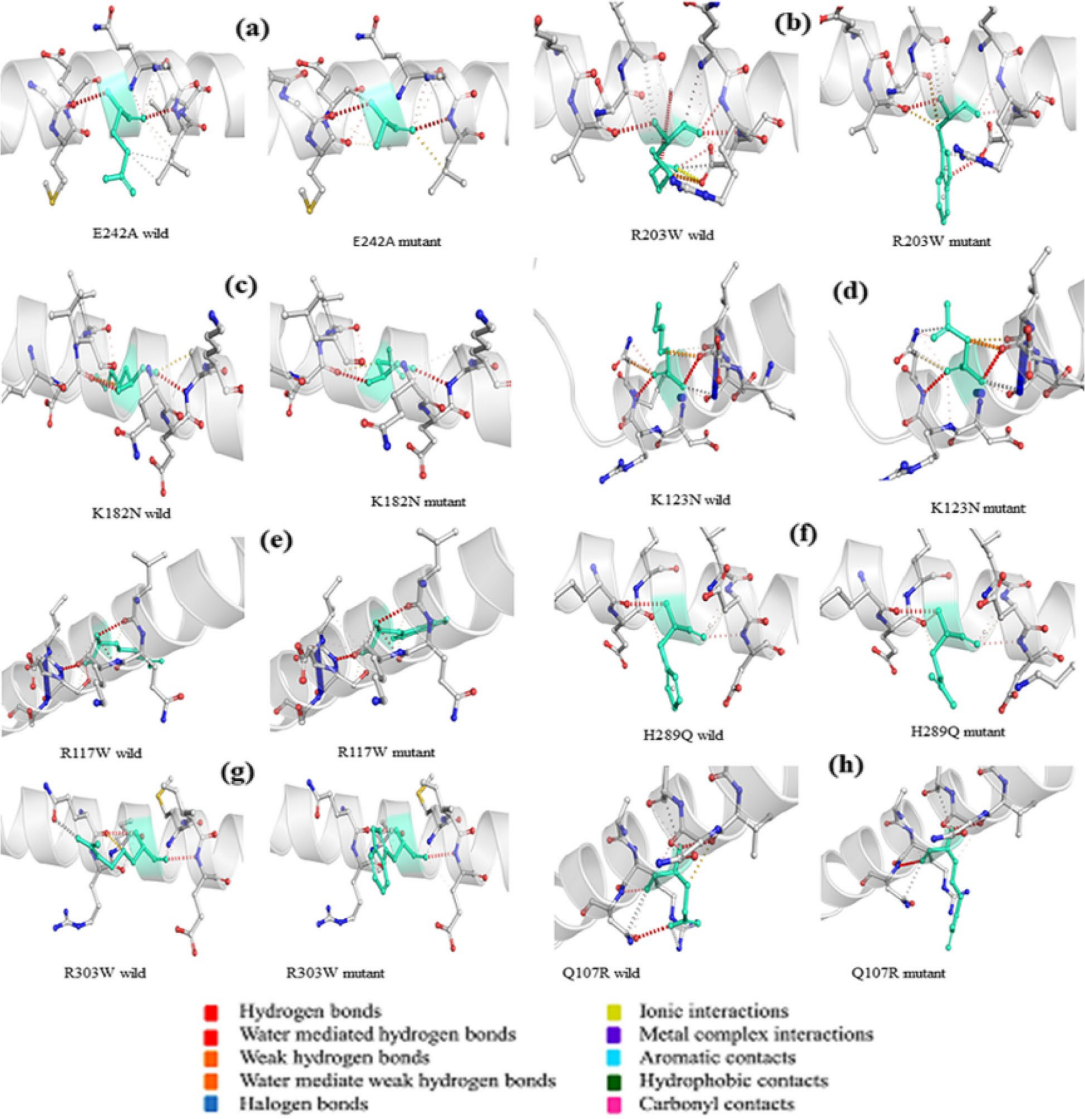
Inter-atomic interaction difference of the wild-type TUFT1 vs the mutants by DynaMut server. Light-green colored native and mutant residues are represented as sticks along with nearby residues participating in the interaction. Interactions like hydrogen bonding and ionic interactions are represented by dot points in various colors

The DynaMut server was used to evaluate the predicted interatomic interactions of eight harmful nsSNPs that were chosen from upstream analyses. The DynaMut server showed the predictions of the ΔΔG and Δ vibrational entropy energy by ENCoM between the mutant and wild-type. According to the predicted DynaMut ΔΔG values, R117W, H289Q, and Q107R were stabilizing the TUFT1 protein when compared to the wild type. The ΔΔG SDM value decreased in E242A, K182N, and K123N when compared to other mutants, and ΔΔS ENCoM showed destabilizing effect for all mutants. Amino acid alterations were detected for all the variants from ΔΔS_Vib_ ENCoM values, indicating enhanced molecular flexibility except for R117W. The prediction from the above server is given in Table 5. The differences in the interatomic interactions such as hydrogen bonds and ionic interactions of the wild-type and the mutant are depicted in Fig. 7.

**Table 5:** Interatomic interaction of mutant residues and native TUFT1

Amino acid change	ΔΔG DynaMut (kcal/mol)	ΔΔG ENCoM (kcal/mol)	ΔΔG mCSM (kcal/mol)	ΔΔG SDM (kcal/mol)	ΔΔG DUET (kcal/mol)	ΔΔS_Vib_ENCoM (kcal.mol^−1^.K^−1^)
E242A	− 0.319 (destabilizing)	− 0.116 (destabilizing)	− 0.634 (destabilizing)	− 0.410 (destabilizing)	− 0.464 (destabilizing)	0.145 (increase of molecule flexibility)
R303W	− 0.283 (destabilizing)	− 0.099 (destabilizing)	− 0.047 (destabilizing)	0.090 (stabilizing)	− 0.211 (destabilizing)	0.124 (increase of molecule flexibility)
K182N	− 0.059 (destabilizing)	− 0.133 (destabilizing)	0.046 (stabilizing)	− 0.800 (destabilizing)	0.174 (stabilizing)	0.166 (increase of molecule flexibility)
K123N	− 0.066 (destabilizing)	− 0.005 (destabilizing)	− 0.055 (destabilizing)	− 0.430 (destabilizing)	0.153 (stabilizing)	0.007 (increase of molecule flexibility)
R117W	0.244 (stabilizing)	0.102 (destabilizing)	− 0.342 (destabilizing)	0.100 (stabilizing)	− 0.408 (destabilizing)	− 0.128 (decrease of molecule flexibility)
H289Q	0.196 (stabilizing)	− 0.097 (destabilizing)	0.726 (stabilizing)	0.250 (stabilizing)	0.869 (stabilizing)	0.121 (increase of molecule flexibility)
R203W	− 0.223 (destabilizing)	− 0.028 (destabilizing)	− 0.423 (destabilizing)	− 0.430 (stabilizing)	− 0.365 (destabilizing)	0.035 (increase of molecule flexibility)
Q107R	0.187 (stabilizing)	− 0.056 (destabilizing)	− 0.099 (destabilizing)	0.050 (stabilizing)	0.251 (stabilizing)	0.069 (increase of molecule flexibility)

## Discussion

Genetic differences between individuals can influence therapeutic response and drug-induced adverse effects in addition to disease susceptibility. Studying the effects of functional exonic SNPs in proteins correlated with the disease can help in developing new drugs to reverse the consequences of such mutations in the population. The current study predicted the consequences of nsSNPs of *TUFT1* using various in silico methods.

The nsSNPs of the *TUFT1* gene were initially determined using sequence-based methods such as SIFT and POLYPHEN and those predicted as deleterious were validated using PROVEAN, SNPs&GO, and PANTHER. The SNPs&GO gives the prediction of both PHD-SNP and PANTHER in addition. Differentiating the scores of all the in silico tools, E242A, R303W, K182N, K123N, R117W, H289Q, R203W, and Q107R, were found to be highly deleterious. Screening the 304 nsSNPs through six in silico tools, eight highly damaging nsSNPs were identified. These eight deleterious nsSNPs include rs4994616, rs148582735, rs149655288, rs149655288, rs150612239, rs369673392, rs370920800, and rs374164451.

The biological mechanism in protein, such as stability or folding, is generally controlled by conserved residues [29]. Enzymatic sites include functional amino acids, which exhibit significant protein–protein interaction [30]. Compared to other residues of TUFT1, these eight nsSNP amino acid residues have a higher degree of conservation. For assessing the deleterious impact, we checked that the amino acid changes in these positions were exposed on the surface of the protein or buried within the protein and the surface accessibility of the residues via the ConSurf web server. The eight amino acid positions are exposed structural residues highlighting their potential impact on interaction with other molecules. Seven variants were evolutionarily conserved indicating their role in protein structural stability except R203W. Six of the eight nsSNPs were found to reduce the stability of TUFT1 revealed by the negative free energy change values as predicted in I-Mutant 3.0. This indicates they may have an impact on the folded structure of the protein. According to literature evidence, both deleterious SNPs and mutations are frequently found in the helix and coil regions and not usually in turns [31]. PSIPRED secondary structure analysis of TUFT1 indicated that the eight high-risk nsSNPs were found to be alpha helixes.

Findings from the Project Hope software have given important details on the potential consequences of missense SNPs of *TUFT1*. The substituted amino acids have various physiochemical characteristics that could damage the structure of the TUFT1 protein. The change in mass and charge of a protein have an impact on the spatial and temporal patterns of protein–protein interactions. The difference in charge by the mutation could cause the mutant residues and their nearby residues to repel one another [32]. As predicted by Project HOPE, the mutant residues E242A, K182N, K123N, and H289Q are smaller in size than the wild-type residues which might interfere with the interaction of other domains that are crucial for the protein’s activity. Compared to wild-type residue, the mutant residue is more hydrophobic in E242A (rs4994616), R303W (rs148582735), R117W (rs150612239), and R203W (rs370920800) SNPs. This could result in the loss of hydrogen bonds with other molecules and might interfere with proper protein folding. From the STRING tool, TUFT1 had direct interactions with ten different proteins, and 5 proteins among 10 were found to be involved in the regulation of tooth and enamel mineralization and odontogenesis suggesting the involvement of TUFT1 in dental fluorosis, dental caries, and amelogenesis imperfect as supported [33]. The functional interaction of other genes in the gene–gene interaction network may be affected by damaging SNPs of the *TUFT1* gene.

With high accuracy, AlphaFold predicts 3D protein structures and generates a predicted local distance difference test (pLDDT) on a range from 0 to 100 that measures confidence for each residue [24]. Based on the local distance difference test C (lDDT-C), pLDDT calculates the degree of the prediction and experimental structure. The DynaMut server gives the change in stability as well as the difference in entropy energy between mutant and wild-type structures. The structural conformation of the TUFT1 protein could be altered by these eight nsSNPs and was found to increase the molecular flexibility of the protein. These structure-based methods for analyzing the impact of mutations on stability offer invaluable information on illness and drug resistance variants and direct protein engineering efforts [34].

Our study explored the *TUFT1* gene polymorphism using various in silico tools. In summary, it can be suggested that these eight SNPs (rs4994616, rs148582735, rs149655288, rs150612239, rs369673392, rs370920800, rs374164451) may affect the TUFT1 protein functions since they are found to be both structurally and functionally deleterious. Accordingly, prioritizing such SNPs for further analysis can be done by systemically analyzing their effects through these types of comprehensive studies. To confirm the deleterious variants of *TUFT1*, further laboratory analysis and in vivo studies are recommended.

## Conclusion

Our in silico SNP study identified eight potential high-risk deleterious nsSNPs of *TUFT1*, and the variants are likely to have an effect on the protein structure and/or function. Further wet lab data and genome association studies are needed to confirm the functional variants to consider as candidate markers in causing oral/dental diseases related to *TUFT1* for diagnosis and therapeutic interventions.

## Data Availability

All data analyzed during this study are included in this article.

## Abbreviations

TUFT1: Tuftelin 1
nsSNPs: Non-synonymous SNPs
SNP: Single-nucleotide polymorphism
AI: Amelogenesis imperfecta
NCBI: National Center for Biotechnology Information
SIFT: Sorting Intolerant from Tolerant
Polyphen-2: Polymorphism phenotyping v2.0
PROVEAN: Protein Variation Effect Analyzer
PANTHER: Protein Analysis through Evolutionary Relationship
SNAP2: Screening for non-acceptable polymorphisms
DAS: Distributed Annotation System
WT: Wild-type
MT: Mutant-type
NMA: Normal mode analysis
ENCoM: Elastic network contact model
ΔΔG: Free energy change
RI: Relative index
TFIP11: Tuftelin-interacting protein 11
AMBN: Ameloblastin
RABGAP1: RabGTPase-activating protein 1
ENAM: Enamelin
AMELX: Amelogenin
RABGAP1L: RabGTPase-activating protein 1-like
MMP20: Matrix metalloproteinase-20
SMC6: Structural maintenance of chromosomes protein 6
DHX15: Pre-mRNA-splicing factor ATP-dependent RNA helicase
ALOX5AP: Arachidonate 5-lipoxygenase-activating protein
pLDDT: Predicted local distance difference test
LDDT-C: Local distance difference test C
